# Molecular Mechanisms of Multi-Organ Failure in COVID-19 and Potential of Stem Cell Therapy

**DOI:** 10.3390/cells10112878

**Published:** 2021-10-25

**Authors:** Aditya Bhalerao, Snehal Raut, Behnam Noorani, Salvatore Mancuso, Luca Cucullo

**Affiliations:** 1 Department of Biological and Biomedical Sciences, Oakland University, Rochester, MI 48309, USA; abhalerao@oakland.edu (A.B.); mancuso@oakland.edu (S.M.); 2 Department of Foundational Medical Studies, Oakland University William Beaumont School of Medicine, Rochester, MI 48309, USA; sraut@oakland.edu; 3 Department of Pharmaceutical Sciences, Jerry H. Hodge School of Pharmacy, Texas Tech University Health Sciences Center, Amarillo, TX 79106, USA; behnam.noorani@ttuhsc.edu

**Keywords:** SARS-CoV-2, COVID-19, stem cells, mesenchymal, therapeutics, regenerative, blood-brain-barrier, oxidative stress

## Abstract

As the number of confirmed cases and deaths occurring from Coronavirus disease 2019 (COVID-19) surges worldwide, health experts are striving hard to fully comprehend the extent of damage caused by the severe acute respiratory syndrome coronavirus 2 (SARS-CoV-2). Although COVID-19 primarily manifests itself in the form of severe respiratory distress, it is also known to cause systemic damage to almost all major organs and organ systems within the body. In this review, we discuss the molecular mechanisms leading to multi-organ failure seen in COVID-19 patients. We also examine the potential of stem cell therapy in treating COVID-19 multi-organ failure cases.

## 1. Introduction

The first outbreak of COVID-19 was reported in Wuhan, China, in December 2019. Subsequently, the outbreak spread globally and was declared a pandemic by the World Health Organization in March 2020 [1]. As of October 2021, over 236 million cases have been reported, with 4.8 million deaths worldwide [2]. The mortality rate of COVID-19 is around 2%, and the disease’s spread is exceedingly high [3,4]. A significant proportion of patients display mild symptoms or are asymptomatic [5]. However, the remainder of the cases are severe, especially in the aged and immune-compromised population [6]. Patients with existing comorbidities such as cardiovascular disease, diabetes mellitus, hypertension, renal dysfunction, liver damage, and cancers exhibit poor prognoses [7]. Death is ultimately caused due to acute respiratory distress syndrome (ARDS), septic shock, cardiac damage, renal dysfunction, and multi-organ failure [8,9].

The host immune response is thought to play a vital role in the pathogenesis and clinical expression of COVID-19. While immune suppression is a risk factor for infection, the immune system’s hyperactivation in response to infection can cause severe complications and organ damage [10]. Though vaccines are in various development stages, testing, and distribution, there are no robust therapeutics for either treatment or symptomatic management of the disease yet. Another alarming fact is that many recovered patients will suffer from lasting effects and disability due to COVID-19 [11]. Therefore, alternative therapeutic modalities need to be studied and developed for managing severe outcomes of the disease. The US Food and Drug Administration (FDA) is currently exploring various single-agent and combination treatments for the disease. These include antivirals, cell and gene therapies, immunomodulators, and neutralizing antibodies [12].

Stem cell-based regenerative medicine is one such field that might hold the key to reviving tissue damage caused by COVID-19. Stem cells play a significant role in developmental biology because they can differentiate into many cell types. Another advantage is that mesenchymal stem cells (MSCs) exhibit immunomodulatory qualities, regulating the immune responses in patients [13]. There are currently several stem cell-based clinical trials in various stages of development, of which quite a few have shown promise in treating patients [14]. Therefore, it is crucial to understand the molecular mechanisms associated with COVID-19 mediated multi-organ damage to efficiently apply stem cell therapy for its treatment [15].

## 2. Current Knowledge

**Figure 1:**
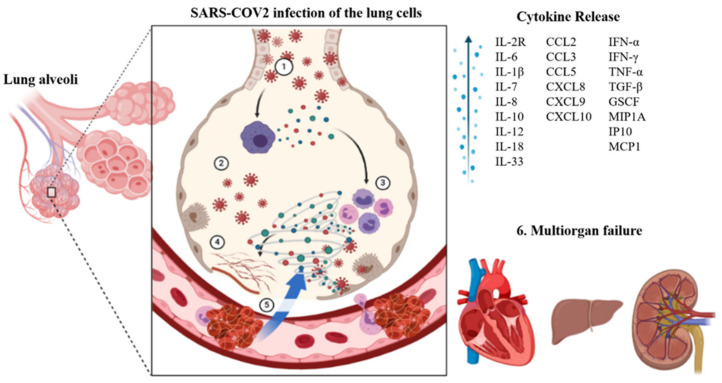
SARS-CoV-2 infection of lungs leading to cytokine storm resulting in multi-organ failure. ① Lung cells infected by the coronavirus, ② Macrophages recognize the virus and release cytokines, ③ Cytokines attract additional immune cells, such as lymphocytes and monocytes, and they generate more cytokines, causing a storm-like cycle of inflammation that damages lung cells. ④ Damage can also occur because of fibrin production (clot formation), ⑤ Blood vessels surrounding the lungs are weakened, allowing fluid to leak into the lung cavities, resulting in respiratory failure, 6. Due to the heightened coagulation state, blood that is meant to flow to other organs such as the heart, liver, and kidneys will be obstructed, causing these organs to fail and lead to multiorgan failure.

The Angiotensin-converting enzyme 2 (ACE2) receptor plays a crucial role in viral entry into the cells [16,17]. This receptor is found on many cell types present throughout the body, primarily the epithelial cells in the lungs [18] and small intestines [19]. The virus uses its spike proteins to bind to the ACE2 receptor and enter the cell [20]. Once inside a cell, the virus uses the cell’s replication machinery to create several viral copies [21]. These infect neighboring cells leading to loss of physiological function. Once the immune system recognizes an infection, it mounts an immune response to fight that infection. This can lead to inflammation in the infected areas. The immune response usually is well regulated and confined to infected regions [22]. However, in the case of COVID-19, we see a hyperactivation of the immune system, whereby it loses the ability to distinguish between self and foreign, therefore causing damage to healthy uninfected tissue. As damage to the tissue builds up, irreparable damage to the organ can occur, causing the organ to fail. This cascade can occur sequentially or in parallel within other organ systems leading to multi-organ failure and death (Figure 1) [23,24,25,26].

### 2.1. Immunological Complications

Cytokines are proteins that recruit immune cells to the site of infection. A moderate immune response leads to a rise in proinflammatory cytokines such as tumor necrosis factor-alpha (TNF-α), interleukin 6 (IL-6), and interleukin 1 (IL-1), and a host of lymphocytes and T cells. However, in a severe immune reaction, we see a sudden extreme induction of these proinflammatory cytokines, also known as the cytokine storm. Cytokine storms lead to widespread inflammation in the body [27]. As a result, vascular membranes become highly permeable, giving rise to fluid movement from blood vessels into organ tissue [28]. The resulting effect is an organ/tissue reaching the brink of failure due to the lack of blood and oxygen. Anti-inflammatory therapies targeting the cytokine storm are suggested to decrease the mortality of COVID-19 patients. Downstream signaling pathways such as JAK/STAT, NF-κB, and NLRP3 as well as cytokine targets such as IL-6, IL-1 β, IFN-γ, TNF-α, IL-12/23, IL-17A, GM-CSF (granulocyte-macrophage colony-stimulating factor) are being explored for treating COVID-19 induced cytokine storm [29].

### 2.2. Hematological Complications

Lymphopenia, or reduction of white blood cells in the blood, is a characteristic clinical hallmark of COVID-19 infection. Typically, CD4, CD8, T, and NK cell counts are significantly decreased. Other observed effects are abnormalities of granulocytes and monocytes along with hypercoagulability leading to thrombocytopenia. An enhanced threat of disseminated intravascular coagulation, specifically, the propensity of clot formation throughout the body, is reported in patients [30,31]. Increased D-dimer concentrations in the non-survivors COVID-19 patients were typical [32,33]. Treatment approaches targeting thrombin, coagulation factor Xa, and thrombin receptor [34] should be considered to reduce SARS-CoV-2 micro thrombosis.

### 2.3. Respiratory Complications

Injury to the lungs in COVID-19 may occur through direct or indirect mechanisms. Various cell types in the airways and lungs exhibit the ACE2 receptor. These include type II alveolar cells, ciliated epithelial cells, and pulmonary vascular endothelium. SARS-CoV-2 can infect these cell types and cause cell death directly. Another mechanism involves activating angiotensin-II, resulting in increased vascular leakiness and pulmonary edema leading to pneumonia [35]. The immune system also plays a critical role in the clinical manifestation of COVID-19 fibroproliferative lung disease [36]. The release of proinflammatory cytokines and activation of macrophages and dendritic cells triggers cell death of infected cells [37,38]. Another factor responsible for pulmonary failure is microthrombi formation in the vasculature of the lungs [39]. Lung biopsies of COVID-19 patients showed an activated complement system in the alveolar epithelial cells with acute and chronic inflammation [38].

### 2.4. Cardiac Complications

Patients with cardiac complications caused due to COVID-19 are termed as suffering from Acute COVID-19 Cardiovascular Syndrome (ACovCS) [40]. There are two different mechanisms for an individual to develop ACovCS. A hypoxia-induced myocardial injury can occur due to a cytokine storm’s precipitation, as discussed before. This is accompanied by intracellular acidosis and increased oxidative stress. On the other hand, myocarditis can occur through ACE2 facilitated direct infection of cardiac myocytes resulting in arrhythmias or cardiac arrest [41,42]. However, it is important to note that in a majority of individuals COVID-19 related myocarditis did not accompany immune cell infiltration pointing to cell death from obstructed blood flow likely due to constricted pericytes or clumping of red blood cells [43]. The histopathological analysis reported fibrosis and myocyte hypertrophy in most COVID-19 patients [44]. Thus, patients with existing cardiovascular disease and hypertension are at heightened risk for mortality due to ischemia and myocardial necrosis factors. Comorbidities such as obesity and diabetes have also been found to indirectly cause adverse cardiac complications. For example, obesity arising out of COVID-19 quarantining is linked with stress, causing a persistent inflammatory state that leads to the deposition of atherosclerotic plaques, rendering obese individuals more susceptible to cardiovascular events [45,46]. A study reported a greater incidence of diabetes in patients with COVID-19 associated cardiac injury [47].

### 2.5. Renal Complications

Patients with renal complications often suffer from acute kidney injury (AKI) and proteinuria. Lymphopenia, macrophage activation syndrome, hypercoagulability, and cytokine storm, among other factors, can promote AKI. Other potential mechanisms can include sepsis, endothelial impairment, and rhabdomyolysis. Furthermore, the microthrombi formation may lead to acute ischemic injury and hypoxia, resulting in renal tubular necrosis [48,49,50]. Various reports confirmed that the development of AKI significantly increased the mortality rate in COVID-19 patients. SARS-CoV-2 infection increased blood urea nitrogen (BUN) and serum creatinine levels, leading to renal failure in many patients [48]. Autopsies of COVID-19 patients detected fibrin thrombi, indicating severe endothelial damage in the glomerular region [51].

### 2.6. Hepatic Complications

Studies have observed that the severity of COVID-19 infection corresponds to the severity of hepatic complications in patients. Very high levels of liver enzymes alanine aminotransferase (ALT) and aspartate aminotransferase (AST) were found in the blood of patients with severe COVID-19 infections [52]. Cytotoxic T cell activation and dysregulated immune responses increased liver biomarkers and COVID-19 severity [53]. One mechanism for viral entry to the liver might be through the ACE2 receptors found on cells lining the bile duct. Other liver injury mechanisms are thought to be linked to oxygen deprivation, the toxicity of antiviral/antimalarial treatments, and inflammatory cells’ passage to the liver [54,55].

### 2.7. Neurological Complications

ACE 2 receptors responsible for attachment and subsequent internalization of SARS-CoV-2 are also found in glial cells in the brain and spinal neurons. Hence, the virus can damage the neuronal tissue and result in hypoxic brain injury and immune-mediated damage to the Central Nervous system (CNS) [56].

A study conducted by the National Hospital, Queen Square, London, and University College London Hospital detailed clinical and paraclinical data on neurological disorders observed during and after the COVID-19 infection. Patients displayed a wide range of CNS and Peripheral Nervous System (PNS) complications together with neuroinflammation. These pathological manifestations can be a direct effect of the virus on the nervous system, para or post-infectious immune-mediated disease, and neurological complications of the systemic impacts of COVID-19 infection. Out of 43 COVID-19 patients, ten patients above the age of 50 suffered septic encephalopathy and presented symptoms such as confusion, psychosis, seizures, etc. Complications such as difficulty speaking, deteriorating vision, cognitive abilities, and Acute Disseminated Encephalomyelitis (ADEM) with hemorrhagic transformations were reported in critically ill patients [57]. Alteration in mental status was frequent in patients with severe infection, especially in those requiring intensive care management. However, this was documented more in older groups, which might be suffering from latent neurocognitive degenerative disease or multiple medical comorbidities, often associated with sepsis and hypoxia. MRI diagnosis in severe cases showed abnormalities in the brain’s temporal lobe with hemorrhagic lesions. Furthermore, features like vascular and ADEM-like pathology, with macrophages and axonal injury, were reported.

Further, seizures were reported with viral encephalitis and subsequent activation of neuro-inflammatory pathways in critically ill patients. Lymphocytic panencephalitis, meningitis, and brainstem inflammatory change with neuronal loss were observed in post-mortem reports [58]. A recent report on neurological complications of children of age < 18 years described a distinct neurological syndrome associated with lesions in the corpus callosum’s splenium. These patients were previously healthy but had the onset of neurological symptoms after the COVID-19 infection. Symptoms observed were encephalopathy, headaches, brainstem-cerebellar signs, muscle weakness, and reduced reflexes [59].

To summarize, the neurological manifestations of COVID-19 can be divided into direct and indirect effects. Symptoms like anosmia, hypogeusia meningitis, encephalitis, cerebral vasculitis, and myalgia result from a direct viral invasion of the CNS and PNS. Encephalopathy arising out of hypoxia, cytokine storm, and hypercoagulable state leading to stroke are some of the indirect manifestations seen in the CNS [60]. Another critical factor that needs to be considered is the impairment of the blood–brain barrier (BBB) due to endothelial dysfunction. This may give rise to the virus’s invasion and inflammatory cells into the CNS, leading to further neurological manifestations [54,61].

## 3. Stem Cell Therapy

Stem cells are undifferentiated or partially differentiated cells in the body that can differentiate into various cell types and proliferate indefinitely (self-renewal). Their primary function is to serve as a reserve for the body [62]. Stem cells can be of embryonic or adult origin. Based on their functionality, stem cells can be grouped into three categories, pluripotent, hematopoietic, and mesenchymal types [63,64,65]. Pluripotent stem cells can differentiate and mature into any of the three fundamental groups of cells important in human developmental biology. Embryonic stem cells are used for in vitro fertilization purposes [66]. Hematopoietic stem cells can differentiate into various types of blood cells. They are obtained from bone marrow or umbilical cord blood and are used in bone marrow transplants. However, both these types of stem cells are currently not used to treat COVID-19. Lastly, MSCs are non-hematopoietic cells that can be differentiated into skeletal tissue such as muscle, bone, cartilage, fat, etc. These cells have immunomodulatory capabilities and have been approved as treatments for a host of autoimmune diseases [67,68]. Additionally, the therapeutic effects of stem cells were recently ascribed to their ability to replace damaged cells. However, we now know that stem cells’ pro-regenerative quality is also due to paracrine functions and the ability to release microvesicles. Microvesicles are known to contain growth factors, bioactive lipids, anti-apoptotic factors which enhance cell function and stimulate angiogenesis in damaged tissues. They are also known to transfer proteins, mRNA, and microRNA between cells [69].

MSCs can be isolated from bone marrow, placenta, umbilical cord blood, and adipose tissue of the same individual (autologous) or another individual (allogeneic) [70]. These are then cultured in vitro and can be injected back into the diseased body. Once inside, they secrete a host of anti-inflammatory mediators that can accelerate tissue repair and revival [71]. MSCs are most likely to have favorable effects when treating acute inflammatory conditions [72]. Conditions frequently encountered in COVID-19 patients, such as cytokine storm, ARDS, and sepsis, are likely to be prime candidates for MSC-based therapy.

### 3.1. Immunomodulatory and Regenerative Effects of MSCs

**Figure 2:**
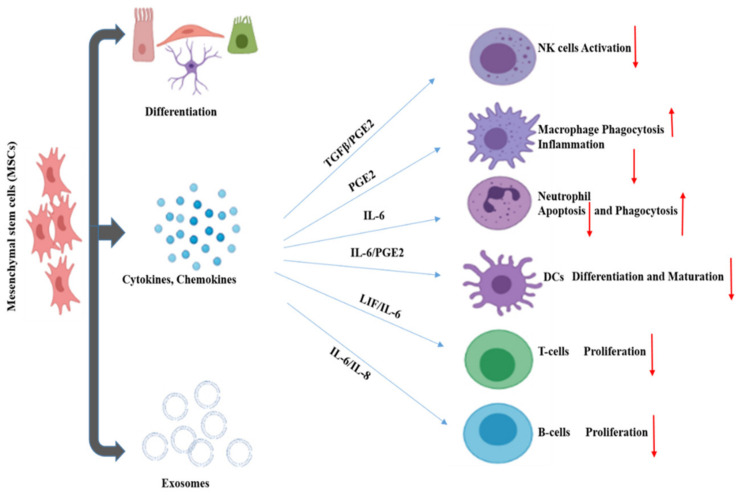
Immunomodulatory effects of mesenchymal stem cells.

MSCs are referred to as “guardians of inflammation” because of their immunomodulatory effect through the secretion of cytokines, chemokines, growth factors, exosomes, etc. MSCs regulate the inflammatory microenvironment through cell-to-cell contact and the secretion of regulatory molecules. These affect the activation, maturation, proliferation, differentiation, and effector functions of various immune cells involved in innate and adaptive immunity. Innate immunity is mediated through NK cells, macrophages, neutrophils. On the other hand, adaptive immunity is facilitated by T cells and B cells (Figure 2). Dendritic cells (DC) act as the connecting link between innate and adaptive immunity.

NK cells secrete cytokines like IFN-γ and exhibit cytotoxic functions in response to viral infection. MSCs can reduce the proliferation of NK cells, inhibiting their cytotoxic functions through key mediators such prostaglandin E2 (PGE2), indolamine 2,3-dioxygenase (IDO), and human leukocyte antigen G5 (HLA-G5) [73]. Macrophages play a vital role in innate immunity by engulfing foreign agents or aberrant cells. There are mainly two forms of activated macrophages grouped into M1 and M2. M1 exhibits a proinflammatory response in contrast to M2, which displays an anti-inflammatory response. PGE2 secreted by MSC influences the macrophage transition from proinflammatory M1 into an anti-inflammatory M2. M2 macrophage expresses high levels of anti-inflammatory cytokines, reduces levels of TNF-α, and IL-12 with higher phagocytic activity [74]. In the inflammatory process, neutrophils generate more reactive oxygen species (ROS) and reduce antioxidant levels. MSCs secrete IL-6, which reduces ROS levels without affecting the phagocytic activity of neutrophils [75]. T-cells, once activated, proliferate and secrete inflammatory cytokines and chemokines. MSCs facilitate their immunomodulatory activity by recruiting local helper (Th) and effector T cells in the inflammatory environment via Chemokine (C-X-C motif) ligands-CXCL9 and CXCL10. B-cells are vital for humoral immunity and secrete antibodies when stimulated. MSCs inhibit B cell activation, proliferation, and differentiation during inflammation through contact inhibition [76,77,78].

MSCs not only play a role in immune regulation, but also the regeneration and reconstruction of tissue. MSCs have differentiation properties conducive to tissue regeneration and can secrete hepatocyte growth factor, vascular endothelial growth factor, and keratinocyte growth factor. These functions can promote the regeneration of type II alveolar epithelial cells [79]. This shows a potential use for MSCs in recovery for severe COVID-19 cases wherein alveolar injury has occurred. It is suggested that MSCs suppress the over-activated inflammatory response, promote recovery of lung function, and potentially influence the progress of pulmonary fibrosis. MSCs have already been shown to significantly contribute to the recovery of patients from severe COVID-19 in Phase 1 clinical trial [14]. A larger phase 2/3 trial is in progress, with 100 participants recruited to evaluate the safety and efficacy of human umbilical cord-derived MSCs as a treatment for severe COVID-19 cases [80]. Additionally, MSCs and other stem cell types and derivatives have been indicated to be capable of promoting regeneration in other tissues, such as vascular [81], renal [82], hepatic [83], and neurological [84]. Through their immune regulatory functions, and role in contributing to tissue repair MSCs represent a promising area for the treatment of COVID-19 [81].

### 3.2. Clinical Trials

The treatment potential of stem cell therapy has been widely explored in immunological, cardiovascular, renal, pulmonary, and hepatic diseases at the preclinical level. However, the current bulk of clinical data is insufficient to demonstrate the unequivocal efficacy of stem cell therapy in patients with complex diseases like organ failure. Patients with dysfunctional renal and hepatic organs are likely to need organ transplants toward their disease’s end-stage. Kidney failure in chronic kidney disease (CKD) or AKI can be attributed to various complicating factors, including diabetes and heart disease. Similarly, liver failure may be caused by multiple factors such as cirrhosis, Hepatitis B or C, and hemochromatosis, etc. However, there are not enough kidney and liver donors available for the number of needing patients. In this backdrop, stem cell therapy has emerged as a promising option for these patients. Studies have shown minimal adverse events and, in some cases, even positive outcomes for patients undergoing stem cell treatments for liver conditions [85,86].

MSC therapies have been approved in several countries for treating several diseases due to their immuno-modulatory effects. They are potent candidates to treat severe cases of COVID-19 and have been used in clinical trials for therapeutic purposes [87]. A list of completed and active clinical trials using MSC therapy to treat COVID-19 associated conditions is provided in Table 1 and Table 2, respectively. Similarly, we have outlined the available results of specific studies in Table 3.

Preclinical and early clinical trials have demonstrated systemic infusions and bronchial instillations as a route of MSC administration for treating ARDS and other respiratory complications. MSC therapy can suppress cytokine storms and lung inflammation by promoting endogenous repair. Adipose-derived MSCs (AD-MSCs), Umbilical cord MSCs (UC-MSCs), bone marrow-derived MSCs (BM-MSCs), and other MSCs, as well as exosomes from MSCs, are being tested in clinical trials. UC-MSCs are the most effective for treating COVID-19 patients due to their proliferative capability and immunomodulatory effects. In a recent clinical study, laboratory tests of C-reactive protein (CRP), alanine aminotransferase (ALT), creatinine, serum ferritin (SF), and platelets before and after the UC-MSCs treatment at days 0, 3, and 7 were recorded for both experimental and control group. In the UC-MSCs treatment group, there was a decline of IL-6 within three days after UC-MSCs infusion, which remained stable for the following four days. The partial pressure of arterial oxygen: percentage of inspired oxygen (PaO2/FiO2) ratio improved in most severe cases. Representative chest CT scan images showed controlled lung lesions within six days, which completely disappeared within two weeks of treatment. A reduced trend in the levels of proinflammatory cytokines was noted within 14 days [104].

In another study, a double-blind, phase 1/2a, randomized, controlled trial was performed in subjects with ARDS secondary to COVID-19. Twenty-four subjects (12 per group) were recruited for this study. At 28 days post the last infusion, patient survival was 91% and 42% in the UC-MSC and control groups, respectively (*p* = 0.015). No serious adverse events were observed related to UC-MSC infusions [137]. Although several studies have been completed, their results have not been declared. As such, several concerns regarding the safety and efficacy of MSCs treatment for COVID-19 associated lung disease are unanswered. For example, we do not know the best administration route, whether intravenous, intramuscular, or through the nasopharyngeal route. Apart from this, there is no consensus on critical factors such as MSC tissue of origin, type of culture environment, and dosing.

## 4. Future Directions

The field of stem cell therapy for treating COVID-19 is gaining a lot of traction. As of October 2021, 100 studies in various countries were displayed on the clinicaltrials.gov website. During a pandemic, as in the case of COVID-19, treatment options are often accelerated and provided emergency authorization to save as many lives as possible. However, it is difficult to ascertain the cause and effect of therapy and outcome based on observational studies alone. Therefore, it is crucial to conduct randomized, double-blinded, placebo-control trials to fully ascertain the efficacy of stem cell therapy as a viable option for COVID-19. Furthermore, it is essential to know the underlying molecular mechanisms through which stem cells fight disease. As such, future studies, both preclinical and clinical with mechanistic approaches, would propel the field further in the right direction.

SARS-CoV-2 attracts leukocytes to the site of infection, thereby initiating the immune response with cytokines’ help. It is known that an increase in ROS accompanies an increase in the immune response. This causes oxidative stress, cell apoptosis, lipid peroxidation, and protein oxidation, further worsening the immune response [139,140]. We hypothesize that facilitating ROS elimination through a better understanding of antioxidant pathways may prevent the oxidative injury caused by SARS-CoV-2 infection. Many studies support the antioxidant and regenerative properties of MSCs [141,142]. A key transcriptional factor, nuclear factor erythroid 2-related factor 2 (NRF2), has been studied for its involvement in regulating antioxidant signals [143]. A recent study published by Olagnier et al. showed suppression of NRF2 in COVID-19 patients, thereby strengthening our hypothesis [144]. Therefore, further studies focusing on NRF2 as a molecular target for treating COVID-19 related complications can provide additional insights into the disease.

## 5. Conclusions

Patients with severe COVID-19 infection often develop multi-organ failure. The damage to organs and organ systems is either through direct infection or hampered physiological processes in response to the infection. It is crucial to consider the immune system as the focal point to understand better and integrate the other organs’ complications. Given the immunomodulatory properties of stem cells, it is essential to conduct further research to study stem cell therapy’s potential in alleviating COVID-19 multi-organ failure.

## Figures and Tables

**Table 1:** List of completed clinical trials studying MSC therapy’s safety and efficacy to treat COVID-19 conditions.

	Title	Interventions	Location	URL (accessed on 15 October 2021)
1	Mesenchymal Stem Cells Therapy in Patients With COVID-19 Pneumonia [88]	Other: Mesenchymal stem cells	Turkey	https://ClinicalTrials.gov/show/NCT04713878
2	A Proof of Concept Study for the DNA Repair Driven by the Mesenchymal Stem Cells in Critical COVID-19 Patients [89]	Biological: Mesenchymal Stem Cells Transplantation	Turkey	https://ClinicalTrials.gov/show/NCT04898088
3	Treatment With Human Umbilical Cord-derived Mesenchymal Stem Cells for Severe Corona Virus Disease 2019 (COVID-19) [80]	Biological: UC-MSCs|Biological: Saline containing 1% Human serum albuminï¼ solution without UC-MSCsï	China	https://ClinicalTrials.gov/show/NCT04288102
4	A Clinical Trial to Determine the Safety and Efficacy of Hope Biosciences Autologous Mesenchymal Stem Cell Therapy (HB-adMSCs) to Provide Protection Against COVID-19 [90]	Biological: HB-adMSCs	United States	https://ClinicalTrials.gov/show/NCT04349631
5	A Randomized, Double-Blind, Placebo-Controlled Clinical Trial to Determine the Safety and Efficacy of Hope Biosciences Allogeneic Mesenchymal Stem Cell Therapy (HB-adMSCs) to Provide Protection Against COVID-19 [91]	Biological: HB-adMSCs|Other: Placebos	United States	https://ClinicalTrials.gov/show/NCT04348435
6	Mesenchymal Stem Cells for the Treatment of COVID-19 [92]	Biological: PrimePro|Other: Placebo	United States	https://ClinicalTrials.gov/show/NCT04573270
7	Treatment of COVID-19 Associated Pneumonia With Allogenic Pooled Olfactory Mucosa-derived Mesenchymal Stem Cells [93]	Biological: Allogenic pooled olfactory mucosa-derived mesenchymal stem cells|Other: Standard treatment according to the Clinical protocols	Belarus	https://ClinicalTrials.gov/show/NCT04382547
8	Use of UC-MSCs for COVID-19 Patients [94]	Biological: Umbilical Cord Mesenchymal Stem Cells + Heparin along with best supportive care.|Other: Vehicle + Heparin along with best supportive care	United States	https://ClinicalTrials.gov/show/NCT04355728
9	An Exploratory Study of ADR-001 in Patients With Severe Pneumonia Caused by SARS-CoV-2 Infection [95]	Biological: Mesenchymal stem cell	Japan	https://ClinicalTrials.gov/show/NCT04522986
10	Therapeutic Study to Evaluate the Safety and Efficacy of DW-MSC in COVID-19 Patients [96]	Drug: allogeneic mesenchymal stem cell|Other: Placebo	Indonesia	https://ClinicalTrials.gov/show/NCT04535856
11	Extracellular Vesicle Infusion Treatment for COVID-19 Associated ARDS [97]	Biological: DB-001|Other: Intravenous normal saline	United States	https://ClinicalTrials.gov/show/NCT04493242
12	A Pilot Clinical Study on Inhalation of Mesenchymal Stem Cells Exosomes Treating Severe Novel Coronavirus Pneumonia [98]	Biological: MSCs-derived exosomes	China	https://ClinicalTrials.gov/show/NCT04276987
13	Investigational Treatments for COVID-19 in Tertiary Care Hospital of Pakistan [99]	Procedure: Therapeutic Plasma exchange|Biological: Convalescent Plasma|Drug: Tocilizumab|Drug: Remdesivir|Biological: Mesenchymal stem cell therapy	Pakistan	https://ClinicalTrials.gov/show/NCT04492501
14	Clinical Use of Stem Cells for the Treatment of COVID-19 [100]	Biological: MSC Treatment|Biological: Saline Control	Turkey	https://ClinicalTrials.gov/show/NCT04392778
15	Evaluation of Safety and Efficiency of Method of Exosome Inhalation in SARS-CoV-2 Associated Pneumonia [101]	Drug: EXO 1 inhalation|Drug: EXO 2 inhalation|Drug: Placebo inhalation	Russian Federation	https://ClinicalTrials.gov/show/NCT04491240
16	Menstrual Blood Stem Cells in Severe COVID-19 [102]	Biological: Allogeneic human menstrual blood stem cells secretome|Other: Intravenous saline injection	Islamic Republic of Iran	https://ClinicalTrials.gov/show/NCT05019287
17	Cellular Immuno-Therapy for COVID-19 Acute Respiratory Distress Syndrome [103]	Biological: Mesenchymal Stromal Cells	Canada	https://ClinicalTrials.gov/show/NCT04400032

**Table 2:** List of active clinical trials studying the safety and efficacy of MSC therapy to treat COVID-19 induced conditions that have completed recruitment as of October 2021.

	Title	Interventions	Locations	URL (accessed on 15 October 2021)
1	Safety and Efficacy of Mesenchymal Stem Cells in the Management of Severe COVID-19 Pneumonia [105]	Biological: Umbilical cord-derived mesenchymal stem cells|Biological: Placebo		https://ClinicalTrials.gov/show/NCT04429763
2	Novel Coronavirus Induced Severe Pneumonia Treated by Dental Pulp Mesenchymal Stem Cells [106]	Biological: Dental pulp mesenchymal stem cells		https://ClinicalTrials.gov/show/NCT04302519
3	NestaCellÂ^®^ Mesenchymal Stem Cell to Treat Patients with Severe COVID-19 Pneumonia [107]	Biological: NestaCellÂ^®^|Biological: Placebo	Brazil	https://ClinicalTrials.gov/show/NCT04315987
4	Safety and Effectiveness of Mesenchymal Stem Cells in the Treatment of Pneumonia of Coronavirus Disease 2019 [108]	Drug: Oseltamivir|Drug: hormones|Device: oxygen therapy|Procedure: mesenchymal stem cells	China	https://ClinicalTrials.gov/show/NCT04371601
5	Bone Marrow-Derived Mesenchymal Stem Cell Treatment for Severe Patients with Coronavirus Disease 2019 (COVID-19) [109]	Biological: BM-MSCs|Biological: Placebo	China	https://ClinicalTrials.gov/show/NCT04346368
6	Use of Mesenchymal Stem Cells in Acute Respiratory Distress Syndrome Caused by COVID-19 [110]	Biological: Mesenchymal Stem Cells derived from Wharton Jelly of Umbilical cords	Mexico	https://ClinicalTrials.gov/show/NCT04456361
7	Study of Allogeneic Adipose-Derived Mesenchymal Stem Cells for Treatment of COVID-19 Acute Respiratory Distress [111]	Biological: COVI-MSC|Drug: Placebo		https://ClinicalTrials.gov/show/NCT04905836
8	Efficacy of Infusions of MSC From Wharton Jelly in the SARS-CoV-2 (COVID-19) Related Acute Respiratory Distress Syndrome [112]	Biological: Ex vivo expanded Wharton’s Jelly Mesenchymal Stem Cells|Biological: Placebo		https://ClinicalTrials.gov/show/NCT04625738
9	Study of Allogeneic Adipose-Derived Mesenchymal Stem Cells to Treat Post COVID-19 “Long Haul” Pulmonary Compromise [113]	Biological: COVI-MSC|Biological: Placebo		https://ClinicalTrials.gov/show/NCT04992247
10	Study of Human Umbilical Cord Mesenchymal Stem Cells in the Treatment of Severe COVID-19 [114]	Biological: UC-MSCs|Drug: Placebo	China	https://ClinicalTrials.gov/show/NCT04273646
11	Clinical Study for Subjects With COVID-19 Using Allogeneic Adipose Tissue-Derived Mesenchymal Stem Cells [115]	Biological: Autologous adipose-derived stem cells		https://ClinicalTrials.gov/show/NCT05017298
12	Expanded Access Protocol on Bone Marrow Mesenchymal Stem Cell Derived Extracellular Vesicle Infusion Treatment for Patients With COVID-19 Associated ARDS [116]	Biological: Bone Marrow Mesenchymal Stem Cell Derived Extracellular Vesicles Infusion Treatment		https://ClinicalTrials.gov/show/NCT04657458
13	Clinical Trial to Assess the Safety and Efficacy of Intravenous Administration of Allogeneic Adult Mesenchymal Stem Cells of Expanded Adipose Tissue in Patients with Severe Pneumonia Due to COVID-19 [117]	Drug: Allogeneic and Expanded Adipose Tissue-Derived Mesenchymal Stem Cells	Spain	https://ClinicalTrials.gov/show/NCT04366323
14	Study to Evaluate the Efficacy and Safety of AstroStem-V in Treatment of COVID-19 Pneumonia [118]	Drug: AstroStem-V		https://ClinicalTrials.gov/show/NCT04527224
15	Autologous Adipose-derived Stem Cells (AdMSCs) for COVID-19 [119]	Biological: autologous adipose-derived stem cells		https://ClinicalTrials.gov/show/NCT04428801
16	Adipose Mesenchymal Cells for Abatement of SARS-CoV-2 Respiratory Compromise in COVID-19 Disease [120]	Biological: Autologous Adipose MSC’s		https://ClinicalTrials.gov/show/NCT04352803
17	Umbilical Cord Tissue (UC) Derived Mesenchymal Stem Cells (MSCs) Versus Placebo to Treat Acute Pulmonary Inflammation Due to COVID-19 [121]	Biological: UCMSCs|Other: Placebo	United States	https://ClinicalTrials.gov/show/NCT04490486
18	Efficacy and Safety Study of Allogeneic HB-adMSCs for the Treatment of COVID-19 [122]	Biological: HB-adMSC|Other: Placebo	United States	https://ClinicalTrials.gov/show/NCT04362189
19	Study of Intravenous COVI-MSC for Treatment of COVID-19-Induced Acute Respiratory Distress [123]	Biological: COVI-MSC|Drug: Placebo		https://ClinicalTrials.gov/show/NCT04903327
20	Mesenchymal Stem Cells (MSCs) in Inflammation-Resolution Programs of Coronavirus Disease 2019 (COVID-19) Induced Acute Respiratory Distress Syndrome (ARDS) [124]	Biological: MSC	Germany	https://ClinicalTrials.gov/show/NCT04377334
21	Study of the Safety of Therapeutic Tx with Immunomodulatory MSC in Adults With COVID-19 Infection Requiring Mechanical Ventilation [125]	Biological: BM-Allo. MSC|Biological: Placebo	United States	https://ClinicalTrials.gov/show/NCT04397796
22	Use of hUC-MSC Product (BX-U001) for the Treatment of COVID-19 With ARDS [126]	Biological: Human umbilical cord mesenchymal stem cells + best supportive care|Other: Placebo control + best supportive care		https://ClinicalTrials.gov/show/NCT04452097
23	The Use of Exosomes for the Treatment of Acute Respiratory Distress Syndrome or Novel Coronavirus Pneumonia Caused by COVID-19 [127]	Drug: MSC-exosomes delivered intravenously every other day on an escalating dose: (2:4:8)|Drug: MSC-exosomes delivered intravenously every other day on an escalating dose (8:4:8)|Drug: MSC-exosomes delivered intravenously every other day (8:8:8)	United States	https://ClinicalTrials.gov/show/NCT04798716
24	Safety and Feasibility of Allogenic MSC in the Treatment of COVID-19 [128]	Biological: Mesenchymal Stromal Cells infusion		https://ClinicalTrials.gov/show/NCT04467047
25	ACT-20 in Patients with Severe COVID-19 Pneumonia [129]	Biological: ACT-20-MSC|Biological: ACT-20-CM|Biological: Placebo		https://ClinicalTrials.gov/show/NCT04398303
26	Repair of Acute Respiratory Distress Syndrome by Stromal Cell Administration (REALIST) (COVID-19) [130]	Biological: Human umbilical cord derived CD362 enriched MSCs|Biological: Placebo (Plasma-Lyte 148)	United Kingdom	https://ClinicalTrials.gov/show/NCT03042143
27	Safety and Efficiency of Method of Exosome Inhalation in COVID-19 Associated Pneumonia [131]	Drug: EXO 1 inhalation|Drug: EXO 2 inhalation|Drug: Placebo inhalation	Russian Federation	https://ClinicalTrials.gov/show/NCT04602442
28	Mesenchymal Stromal Cell Therapy for The Treatment of Acute Respiratory Distress Syndrome [132]	Drug: Mesenchymal Stromal Stem Cells-KI-MSC-PL-205	Sweden	https://ClinicalTrials.gov/show/NCT04447833
29	MSCs in COVID-19 ARDS [133]	Biological: Remestemcel-L|Drug: Placebo	United States	https://ClinicalTrials.gov/show/NCT04371393
30	Cell Therapy Using Umbilical Cord-derived Mesenchymal Stromal Cells in SARS-CoV-2-related ARDS [134]	Biological: Umbilical cord Wharton’s jelly derived human|Other: NaCl 0.9%	France	https://ClinicalTrials.gov/show/NCT04333368
31	Stem Cell Educator Therapy Treat the Viral Inflammation in COVID-19 [135]	Combination Product: Stem Cell Educator-Treated Mononuclear Cells Apheresis		https://ClinicalTrials.gov/show/NCT04299152
32	Multiple Dosing of Mesenchymal Stromal Cells in Patients with ARDS (COVID-19) [136]	Biological: Mesenchymal stromal cells|Other: Placebo	United States	https://ClinicalTrials.gov/show/NCT04466098

**Table 3:** Results of completed clinical trials studying MSC therapy to treat COVID-19 conditions.

Therapy	Side effects of therapy	Results	No. of Patients
Exosomes (ExoFlo)-allogeneic-bone marrow-derived-mesenchymal stem cells [138]	Hypoxic respiratory failure, pulmonary embolism, acute renal failure, and expiration.	Improved PaO2/FiO2 ratio. Substantial reduction in levels of the CRP, ferritin, acute phase reactants, and D-dimer. Reductions in neutrophil count. Increase in lymphocyte count, including subsets staining positive for CD3^+^, CD4^+^, CD8^+^.	24

## Abbreviations

ACE2	Angiotensin-Converting Enzyme 2
ACovCS	Acute COVID-19 Cardiovascular Syndrome
AKI	Acute Kidney Injury
ALT	Alanine Aminotransferase
ARDS	Acute Respiratory Distress Syndrome
AST	Aspartate Aminotransferase
BBB	Blood–brain barrier
BUN	Blood Urea Nitrogen
CD4	Cluster of Differentiation 4
CD8	Cluster of Differentiation 8
CKD	Chronic Kidney Disease
CNS	Central Nervous System
COVID-19	Coronavirus Disease 2019
DIC	Disseminated Intravascular Coagulation
FDA	Food and Drug Administration
FiO_2_	Percentage of inspired oxygen
IFN-γ	Interferon Gamma
IL-1	Interleukin 1
IL-1RA	Interleukin Receptor type 1
IL-6	Interleukin 6
IP-10	Interferon Inducible Cytokine IP10
MCP-1	Monocyte Chemoattractant Protein 1
MIP-1	Macrophage Inflammatory Protein 1-α
MSCs	Mesenchymal Stem Cells
NK	Natural Killer
NRF2	Nuclear factor erythroid 2-Related Factor 2
PaO_2_	Partial Pressure of arterial oxygen
PNS	Peripheral Nervous System
ROS	Reactive oxygen species
SARS-CoV-2	Severe Acute Respiratory Syndrome CoronaVirus 2
TNF-α	Tumor Necrosis Factor-alpha
